# Pre-Diagnosis Recreational Physical Activity and Lung Cancer Mortality within the California Teachers Study

**DOI:** 10.21203/rs.3.rs-8138657/v1

**Published:** 2025-12-03

**Authors:** Emily L. Cauble, Mia Blanchard, Peggy Reynolds, Emma S. Spielfogel, Jessica Clague DeHart

**Affiliations:** City of Hope; Claremont Graduate University; University of California San Francisco; City of Hope; Claremont Graduate University

**Keywords:** Lung cancer, Survivorship, Physical activity, Risk factors, Cohort study

## Abstract

**Purpose:**

Although physical activity (PA) levels have been linked to decreased lung cancer mortality, the magnitude of associations and delineation of biological and behavioral risk factors is often inconsistent. Our study aims to address this gap by elucidating the associations of lung cancer mortality with time-varying and exertion-varying pre-diagnosis PA levels.

**Methods:**

We examined the associations between PA and lung cancer mortality among 1,768 women enrolled in the California Teachers Study cohort and diagnosed with lung cancer between 1995–2019. Pre-diagnosis lifetime and recent PA were assessed. Multivariable Cox regressions provided hazard ratio (HR) and 95% confidence interval (CI) estimates.

**Results:**

Similar risks of lung cancer mortality were observed across all PA variables. Ever and/or former smokers who engaged in higher levels of moderate, lifetime PA had a lower risk of lung cancer mortality. Ever and/or current smokers who engaged in intermediate to high levels of strenuous, lifetime PA had increased risk of lung cancer mortality, while never smokers saw a protective effect on lung cancer mortality.

**Conclusion:**

The results of this study suggest that smoking significantly modifies the association between PA and lung cancer mortality. Although the mechanisms underlying these findings remain unclear, we hypothesize that excessive strenuous PA among ever and/or current smokers exacerbates the inflammatory damage already induced by smoke exposure, compromising immune cell recovery and leading to reduced lung cancer survival in this group.

## Introduction

According to the American Cancer Society, lung cancer is the 2nd most common cancer in women the United States, and leading cause of cancer death [1]. Although incidence and mortality rates have decreased over time due to advancements in treatment and early detection, the number of lung cancer-related deaths remains substantial; women have also been shown to have smaller decreases in incidence compared men (8% vs 14% decrease) [1–4]. In 2024, it was estimated that lung cancer will account for over 125,000 deaths in the United States [1–3].

The most common form of lung cancer, non-small cell lung cancer (NSCLC), is often diagnosed at an advanced stage [1, 5]. NSCLC accounts for 80–85% of lung cancer cases, and even with treatment, has a 5-year survival rate of only 28%; specifically, women have a 31.3% 5-year survival estimate [6, 7]. The 5-year survival rate for small cell lung cancer is even lower at 7% [6]. Successful treatment depends on disease severity, and while systemic therapy modestly prolongs survival in patients with advanced lung cancer, tumors in some patients are highly resistant to therapy [5, 8]. This grave picture highlights the crucial necessity to investigate biological mechanisms and identify factors that may be used to develop interventions, tailor treatment regimens, and improve lung cancer prognosis.

Smoking is widely recognized as a primary risk factor for lung cancer development and progression for both men and women, with a substantial body of literature reporting that smokers have an increased risk for developing and dying from lung cancer compared to non-smokers [9, 10]. Research examining the biological mechanisms of this association has demonstrated that smokers have elevated levels of various immune markers (e.g., increased white blood cell counts and pro-inflammatory markers), as well as increased oxidative stress compared to non-smokers [11]. Smoking promotes chronic, systemic inflammation by directly impacting epithelial and immune cells within the airway (via the oral and nasal cavities), releasing various pro-inflammatory immune markers and activating additional immune cells [9, 12].

To help prevent lung cancer development and improve prognoses, healthier lifestyle choices are often encouraged, including physical activity (PA) and smoking cessation. Although PA levels have been linked to decreased lung cancer risk and mortality in previous epidemiological studies, these studies differ greatly in the magnitude of significant associations and inconsistently delineate by biological sex at birth, smoking status, PA levels, and lifetime PA leading up to a lung cancer diagnosis [13–22]. Our study aims to further elucidate the associations of lung cancer mortality with time-varying and exertion-varying PA levels and to address the inconsistent associations presented in previous epidemiological studies.

## Methods

### Study Population and Data Collection

The California Teacher Study (CTS) cohort was established in 1995–1996 and consists of 133,477 active and retired female teachers and administrators in California. The cohort has been previously described [23], and participants provided informed consent at baseline. This project was approved by the Institutional Review Board of Claremont Graduate University. All methods were executed in accordance with relevant institutional and national guidelines. Due to standard cohort exclusions (i.e., consenting to breast cancer research only, moving out of California before completion of the baseline survey, invalid baseline surveys due to missing data, participant death before the return of the baseline survey, person(s) other than an identified proxy completing the baseline survey, or follow-up questionnaire was completed prior to the baseline survey), the starting eligible population for this cohort study was 125,120 women. Study start date was the date that each participant completed the baseline questionnaire (late 1990’s). The following additional exclusions were applied to arrive at a final analytical cohort: participants whose start age was the same as their end age were censored (no follow-up) (N = 64), lung cancer incidence before start date (N = 188), incomplete smoke exposure data (N = 841), lung cancer was not classified as malignant (invasive) in both ICD-O-3 and ICD-O-2 (N = 6), incomplete PA data (N = 857), and incomplete alcohol consumption data (N = 6,061). A total of 1,768 participants (including 516 never smokers) who were diagnosed with lung cancer between the time of joining the cohort to the end of 2019 were deemed eligible and are included in our analyses. Of the eligible cohort, 1,043 women died of lung cancer during the study period.

At the start of the study follow-up period, participants submitted a baseline questionnaire that covered extensive demographic and personal information, including PA levels (lifetime PA [from high school through age 54 years] and recent PA [in the three years before joining the cohort]), recent and past hormonal therapy use, menopausal status, smoking status/exposure, etc. The CTS cohort is linked annually with the California Cancer Registry (CCR) and the California Department of Public Health (CDPH) to ascertain cancer diagnoses and tumor information, as well as date and cause of death in cohort members, respectively.

### Participant covariates

Covariates collected at baseline and considered for our analysis have been associated with lung cancer mortality in previous studies [15, 16, 19]. These covariates include age, race/ethnicity (Non-Hispanic White and Other), first-degree family history of lung cancer (parent, sibling, or child: yes, no, and adopted/not provided), body mass index (BMI) calculated from collected weight and height variables (BMI; <18 kg/m^2^, 18–24 kg/m^2^, 25–29 kg/m^2^, ≥ 30 kg/m^2^), education level (less than high school, technical/high school diploma, associate degree/some college, and university or higher [graduated]), and alcohol consumption (none, < 20grams/day, or ≥20grams/day). Menopausal status (premenopausal, perimenopausal, and postmenopausal) was collected at baseline and derived from responses about menstrual periods; additional data were collected for duration and timing of estrogen and progestin therapy and ages at reported reproductive organ surgeries, if relevant.

Participants also) provided detailed information regarding active and passive smoking history. Respondents were asked if they had ever smoked 100 or more cigarettes during their lifetime and, if so, when they started and stopped smoking. Information on smoking history was also collected, including total lifetime smoking pack years, the presence of household passive smoke exposure, and years since quitting for former smokers. A derived smoking variable was generated that incorporated smoking status and total pack years and was defined as the following levels: never smokers (no pack years), former smokers who had low pack years (≤ median pack years for former smokers), former smokers who had high pack years (> median pack years for former smokers), current smokers who had low pack years (≤ median pack years for current smokers), and current smokers who had high pack years (> median pack years for current smokers).

### Physical Activity Variables

Participants provided detailed information on the baseline questionnaire regarding recreational PA across various periods of their lives (while in high school; between the ages of 18 and 24, 25 and 34, 35 and 44, and 45 and 54 years; as well as during the 3 years before completing the questionnaire). For each time interval, they were asked to indicate the average amount of time spent participating in all moderate activities (e.g., brisk walking, recreational tennis, volleyball, golf, softball, and cycling on level street) and in all strenuous activities (e.g., swimming laps, aerobics, calisthenics, running, jogging, cycling on hills, and racquetball). Participants reported the average number of hours per week (categories: none, 0.5, 1, 1.5, 2, 3, 4–6, 7–10, and ≥ 11 hours) and months per year (categories: 1–3, 4–6, 7–9, and 10–12 months) they engaged in moderate and strenuous PA. For each time interval, separate “hours per week” variables were created for strenuous and moderate PA by multiplying the hours spent per week by the portion of the year in which the woman engaged in the activity.

Lifetime PA was calculated for each participant by multiplying the average hours per week per year (h/wk/y) of activity performed during one of the time periods by the number of years of the relevant time interval and then summing across all time periods. The cumulative measure was divided by the total number of years spent in all the time periods, to provide an average annual lifetime (beginning with high school through current age if < 55 years at baseline) or quasi-average annual lifetime (if 55 years or older at baseline) measure of PA for each woman. A woman’s PA during the three years before completing the baseline questionnaire (recent activity) was also assessed. Each PA variable (moderate and strenuous for lifetime and recent activity) was categorized as tertiles for further analysis (descriptive statistics presented in Online Resource 1). Additionally, a combined PA variable (moderate + strenuous activity, presented as tertiles) was created for both lifetime and recent activity (Online Resource 1).

### Statistical Analyses

Descriptive analyses were conducted to characterize the study population. Multivariable-adjusted hazards ratios (HR) and 95% confidence intervals (CI) for lung cancer mortality associated with PA were obtained by fitting Cox proportional hazards regression models using age as a timescale (where subjects enter at the age they were diagnosed with lung cancer and exit at their event/censoring age). Using age as the time metric ensures that women of the same age are compared and, therefore, controls for differences in the risk of death due to age alone. The first analytical model (Model 1) included adjusting only for respective PA variables as appropriate (e.g., when assessing the mortality risk for moderate lifetime PA, the model was adjusted for age at diagnosis and strenuous lifetime PA). This approach allows for the association for the specific PA variable to be assessed without any confounding effects from the other PA variables (e.g., strenuous activity can be assessed irrespective of the impact of moderate activity). The second model (Model 2) expanded upon Model 1, with additional adjustments for menopause status with hormonal therapy use, smoking total pack-years by smoking status, and alcohol consumption. Sensitivity analyses were performed for additional covariates of interest, including BMI, education level, passive smoke exposure, smoking quit-years, and disease stage. Additionally, detailed treatment data were not available; however, since lung cancer treatment is based greatly on tumor stage, stage at diagnosis was used as a proxy in our statistical models

Follow-up time was calculated as the number of days between the lung cancer diagnosis and either date of death, date participant moved out of California, or end of study date (December 31, 2019), whichever came first. Women who moved out of California, died from a cause other than lung cancer, or did not have an event before December 31, 2019, were censored and contributed person-days to the analysis only up to the date of the respective event.

Stratified Cox proportional hazards analyses were conducted to further assess potential effect modification by smoking status (never, ever, former, and current smoker). Kaplan-Meier curves and log-rank tests were used to examine the differences in survival by level of PA and are based on time since diagnosis, not age at diagnosis. All statistical analyses were performed using SAS^®^ software, version 9.4 [24] and were performed in the secure CTS platform [25].

## Results

The mean follow-up period from baseline to the date of diagnosis was 15 years. The average age at diagnosis was 74.7 (± 9.9) years (Table 1). The majority of women were diagnosed with NSCLC (93.2%) and nearly half presented with distant metastases (49.7%). Premenopausal women were more likely to have higher levels of combined (moderate + strenuous), lifetime PA (42.9%) and lower levels of combined, recent PA (37.3%). While current smokers were more likely to have lower levels of combined, recent PA (43.3%), former smokers were more likely to have higher levels of combined, recent PA (37.2%). Women with a BMI ≥ 30 kg/m3 had lower levels of combined, recent PA (51%).

**Figure 1:**
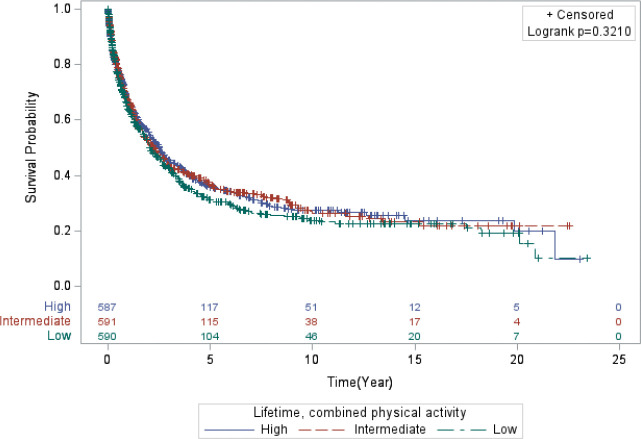
Kaplan-Meier curve displaying the survival time of the women in the study who engaged in lifetime, combined physical activity.

**Figure 2:**
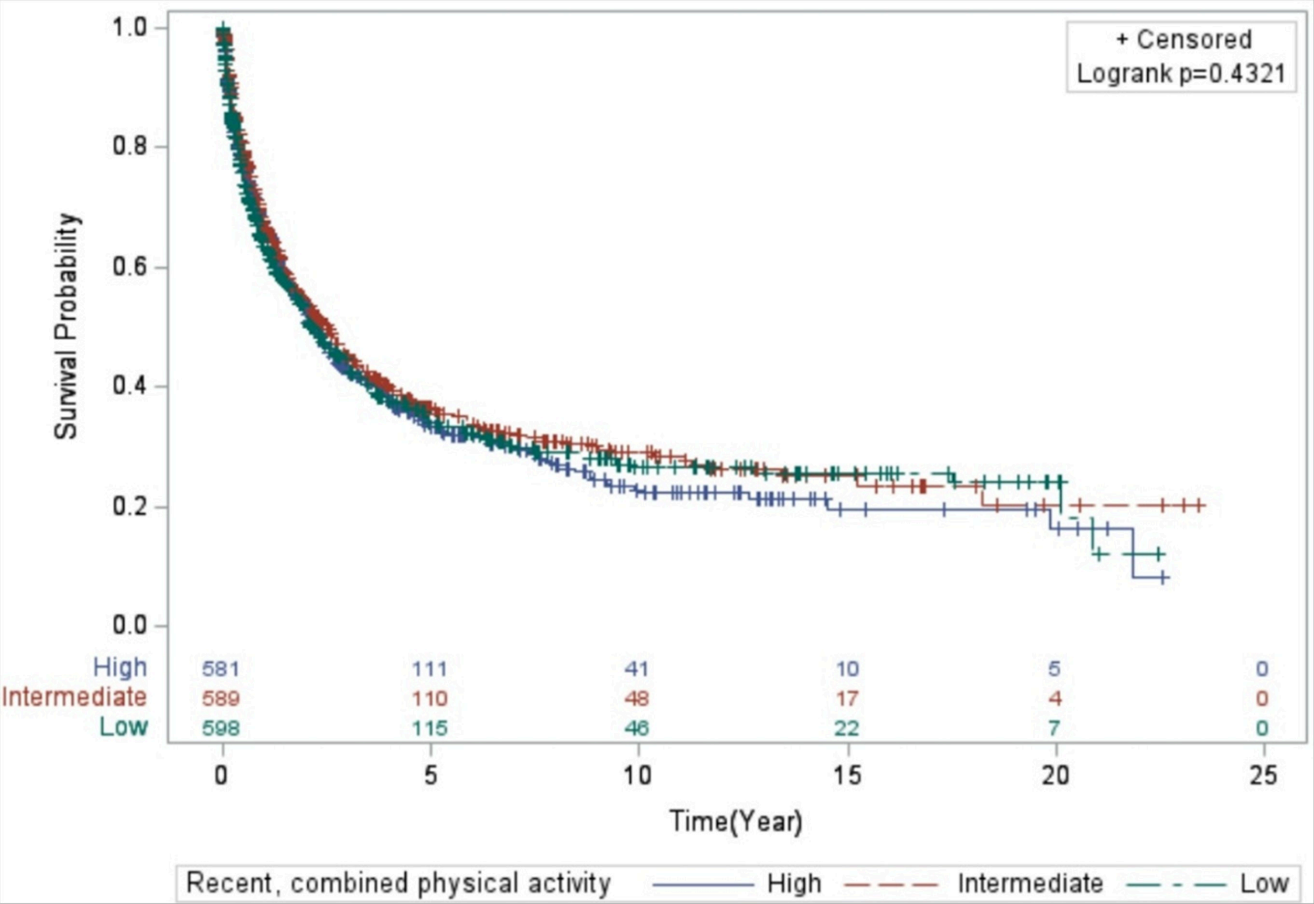
Kaplan-Meier curve displaying the survival time of the women in the study who engaged in recent, combined physical activity

The results (Model 1 and Model 2) for the associations between all PA variables and lung cancer mortality are presented in Table 2. Overall, the results for lung cancer mortality were relatively unremarkable across all PA variables for lifetime and recent activity. Women who reported higher levels of combined, lifetime PA level had the highest median survival time (MST) of 2.5 years compared to 2.1 years for women who reported lower activity levels and 2.3 years for women who reported intermediate levels (*Log-rank p-value*: 0.321; Fig. 1). In contrast, for combined, recent PA, those with intermediate levels of activity survived longer (MST = 2.5 years) compared to those with lower (MST = 2.2) and higher combined, recent PA levels (MST = 2.2 years) (*Log-rank p-value*: 0.4321; Fig. 2).

Associations stratified by smoking status are presented in Table 3. Ever smokers (HR = 0.77, 95%CI = 0.64–0.94) and former smokers (HR = 0.63, 95%CI = 0.49–0.82) who engaged in higher levels of moderate, lifetime PA had a decreased risk of lung cancer mortality. Ever smokers who engaged in intermediate (HR = 1.24, 95%CI = 1.04–1.48) and higher (HR = 1.22, 95%CI = 1.00–1.49) levels of strenuous, lifetime PA saw increased risk of lung cancer mortality. Similarly, current smokers who engaged in intermediate levels of strenuous, lifetime PA also saw increased risk of lung cancer mortality (HR = 1.39, 95%CI = 1.04–1.87). In contrast, never smokers who engaged in higher levels of strenuous, lifetime PA saw a decrease in lung cancer mortality risk (HR = 0.69, 95%CI = 0.49–0.97). Additionally, never smokers saw an increased risk of lung cancer mortality with intermediate levels of strenuous, recent PA (HR = 1.62, 95%CI = 1.12–2.34).

Sensitivity analyses were performed by adjusting for BMI, education level, disease stage (local vs non-local lung cancer, smoking quit-years (among ever and former smokers only), and passive smoke exposure (among never smokers). Inclusion of disease stage in the models attenuated the notable associations, except for the association of increased mortality risk among never smokers who engaged in strenuous, recent activity. Disease stage may act as a mediator in the relationship between PA levels and lung cancer mortality, hence was not included in the final models so that the total effect was not obscured. Additionally, we found that including smoking quit-years did not alter any model results except for ever smokers who engaged in strenuous, lifetime PA, suggesting that quit-years may act as a confounding variable for this specific relationship.

## Discussion

We assessed associations between PA and lung cancer mortality among 1,768 women diagnosed with lung cancer and enrolled in the California Teachers Study from 1995–2019. We highlight the following notable associations from this study: 1) women who have smoked (ever and/or former) and engaged in higher levels of moderate, lifetime PA had a lower risk of lung cancer mortality, 2) ever and/or current smokers who engaged in intermediate to high levels of strenuous, lifetime PA saw an increased risk for lung cancer mortality, while never smokers saw a protective effect with higher levels of strenuous, lifetime PA, and 3) never smokers who engaged in strenuous, recent PA had increased lung CA mortality risk.

While a relatively small number of studies have examined PA and lung cancer-specific mortality, fewer still have explored this association while also stratifying by smoking status, a key determinant of both incidence and mortality in lung cancer. Among those that do not stratify by smoking status, increased PA levels were consistently associated with reduced lung cancer mortality [14, 20]. We found only three studies to date that examine PA and lung cancer mortality risk across smoke exposure groups in women [15, 15, 26]. However, these studies either assess recent PA only or do not account for both duration and exertion of PA, limiting the comparability of their findings to those of the present study.

PA is known to support immune cell infiltration, reduce cancer cell growth and survival, improve the tumor microenvironment, reduce oxidative stress and increase DNA repair processes [27]. Despite these benefits, several studies suggest that PA’s therapeutic effects persist to an extent, while excessive strenuous PA may have an opposite and deleterious effect on lung cancer mortality [16, 19, 28]. Exercise immunology research indicates that high-intensive PA without adequate recovery can trigger immunosuppressive effects that temporarily increase the risk of illness [29, 30]. Specifically, it is thought that following strenuous PA, antibody production as well as circulating levels of lymphocytes and natural killer cells significantly decrease [29, 31, 32]. While more research is warranted to confirm our findings, this temporary immunosuppressive state may offer some explanation for the increased lung cancer mortality risk we observed among never-smoking women who engaged in strenuous, recent PA. This explanation is also consistent with the increased mortality risks we observed in women with a history of smoking, especially in light of the chronic respiratory strain and low-grade inflammation induced by smoking. The deleterious effects of smoke exposure are known to weaken the immune system for years after cessation [9]. These effects, when coupled with the immune impairment precipitated by elevated strenuous PA, may thus account for the elevated lung cancer mortality risks we observed among ever and/or current smokers who engaged in higher levels of strenuous, lifetime PA. Notably, women in our study who were not subject to the inflammatory effects of smoke exposure (never smokers) saw protective effects for lung cancer survival with this same level of increased, lifetime strenuous PA.

Moreover, former and ever smokers who engaged in high levels of moderate lifetime PA also saw protective effects for lung cancer mortality, highlighting the importance of quality and time spent in PA recovery. Moderate, lifetime PA, such as brisk walking or playing golf, may allow for cell recovery and immune repair necessary to mitigate the deleterious effects of smoking, while high levels of strenuous PA, like running or cycling uphill, may not; an observation that is supported in the literature [9, 15].

The limited research on this topic has largely focused on recent, pre-lung cancer diagnosis PA. Interestingly, our stratified analyses revealed a stark contrast in lung cancer mortality risks between lifetime PA and recent PA groups, suggesting that PA and smoking behaviors from high school through adulthood may play a more prominent role in women’s lung cancer survival than in the several years preceding diagnosis. Further studies examining lifetime and exertion-varying PA variables and lung cancer mortality are warranted.

Our findings ultimately expand on those of previous studies with the following arguments about PA and lung cancer mortality prevention: 1) PA regimens (e.g., PA intensity and duration) should be determined through a personalized approach that considers factors like disease stage, physical fitness, smoking status, and other relevant health behaviors; 2) PA interventions aimed at lung cancer mortality prevention should target at-risk individuals as early as is feasible [18], and 3) interventions and public/clinical health promotion efforts should consider that high levels of strenuous PA among those with a history of smoking may not benefit lung cancer prognosis.

The strengths of this study include its prospective design, recent and length of follow-up time (up to 2019), detailed lifetime and recent PA information categorized by exertion level, and extensive data regarding potential confounding factors (e.g., total smoking pack-years and passive household smoke exposure). There are limitations to this study, including 1) PA measures were collected at baseline, with a varying number of years preceding diagnosis, 2) detailed information regarding specific types of PA performed during each time period, which would be needed to calculate MET-hours/week, were not collected, 3) the smoking variables included in this study were collected at baseline, are restricted to cigarette smoking and do not reflect smoking cessation during follow-up, and 4) our study was based in California, a state reported to have high pollution levels, which have previously been associated with lung cancer risk [33, 34]. Our study population is restricted to women only, potentially reducing generalizability; however, postmenopausal women are seldom the focus of studies on lung cancer mortality and PA levels, despite lung cancer remaining the second most common cancer in women, secondary to breast cancer [1]. Given the potential for clinical significance if our findings are confirmed in future studies, further investigation is warranted.

## Figures and Tables

**Figure 5:**
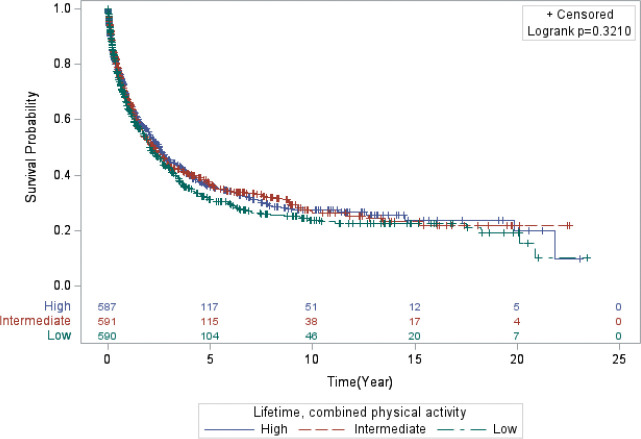
Kaplan-Meier curve displaying the survival time of the women in the study who engaged in lifetime, combined physical activity.

**Table 1:** Baseline participant characteristics among 1,768 women diagnosed with lung cancer in the California Teachers Study stratified by pre-diagnosis physical activity levels (presented as tertiles).

		Lifetime physical activity^a^			Recent physical activity^a,b^		
Characteristic	N (%)	Low	Intermediate	High	Low	Intermediate	High
**No. invasive lung cancer cases**	1768	590	591	587	598	589	581
**Mean age at diagnosis ± SD**		75.6 ± 9.4	74.4 ± 9.7	74.1 ± 10.5	74.7 ± 10	74.1 ± 9.8	75.2 ± 9.8
**Race/ethnicity (%)**							
Non-Hispanic White	1569 (88.7%)	33.1	33.1	33.8	32.4	33.8	33.8
Other^c^	199 (11.3%)	35.2	36.2	28.6	45.2	29.7	25.1
**Socioeconomic status (%)**							
Low SES	68 (3.9%)	30.9	41.2	27.9	39.7	35.3	25.0
2nd quartile	275 (15.6%)	35.6	28.7	35.6	43.3	27.3	29.5
3rd quartile	515 (29.1%)	36.3	31.8	31.8	35.3	32.2	32.4
High SES	900 (50.9%)	31.2	34.9	33.9	29.8	35.3	34.9
**Menopausal status (%)**							
Premenopausal	177 (10%)	22.0	35.0	42.9	37.3	33.9	28.8
Postmenopausal							
No hormone therapy use	391 (22.1%)	34.0	32.5	33.5	35.3	31.0	33.8
Current/former hormone therapy use	975 (55.2%)	34.8	33.5	31.7	32.1	33.0	34.9
All Other	225 (12.7%)	35.1	33.3	31.6	36.0	38.2	25.8
**Smoking status (%)**							
Never smoker	516 (29.2%)	36.1	31.4	32.6	32.2	36.8	31.0
Former smoker	822 (46.5%)	32.5	35.3	32.2	29.9	32.9	37.2
Current smoker	430 (24.3%)	31.9	32.3	35.8	43.3	30.0	26.7
Mean pack-years ± SD		35.8 ± 24.3	30.9 ± 21.9	32.9 ± 22.5	37.9 ± 25.6	30.8 ± 21.7	30.5 ± 20.5
**Body mass index, kg/m**^**2**^ **(%)**							
<18	129 (7.3%)	42.6	30.2	27.1	37.2	34.9	27.9
18–24	963 (54.5%)	32.6	34.4	33.0	28.7	33.0	38.3
25–30	476 (26.9%)	31.7	33.6	34.7	35.9	35.1	29.0
≥30	200 (11.3%)	35.0	30.5	34.5	51.5	29.5	19.0
**Cancer Histology (%)**							
Non-Small Cell Lung Cancer	1647 (93.2%)	33.0	33.9	33.1	33.2	33.5	33.3
Small Cell Lung Cancer	121 (6.8%)	38.0	27.3	34.7	43.0	30.6	26.5
**Stage (%)**							
Localized	455 (25.7%)	28.8	36.9	34.3	32.3	35.2	32.5
Regional extension only	123 (7%)	30.9	37.4	31.7	29.3	33.3	37.4
Regional lymph nodes only	134 (7.6%)	37.3	31.3	31.3	38.1	35.1	26.9
Regional extension and lymph nodes	87 (4.9%)	31.0	29.9	39.1	32.2	29.9	37.9
Distant	879 (49.7%)	35.4	31.4	33.2	33.9	32.9	33.2

a Lifetime and recent categories include physical activity presented as tertiles

b Recent includes PA reported within 3 years prior to baseline

c The “Other” category for race/ethnicity includes Hispanic, Native American, Asian/Pacific Islander, Mixed and Unknown

**Table 2:** Multivariable hazard ratios (HR) and 95% confidence intervals (CI) for the association between physical activity (measured in tertiles) and mortality among 1,768 women diagnosed with invasive lung cancer following enrollment in the California Teachers Study.

	N Cases	N Deaths	Model 1^a^	Model 2^b^
			HR (95% CI)	HR (95% CI)
**Lifetime physical activity**				
**Moderate**				
Low	611	370	1.00 (Reference)	1.00 (Reference)
Intermediate	571	331	0.96 (0.82–1.11)	0.96 (0.82–1.12)
High	586	342	0.87 (0.74–1.03)	0.87 (0.74–1.03)
P for trend			0.1017	0.1214
**Strenuous**				
Low	590	353	1.00 (Reference)	1.00 (Reference)
Intermediate	589	343	1.01 (0.87–1.17)	1.09 (0.94–1.27)
High	589	347	1.00 (0.84–1.18)	1.06 (0.90–1.26)
P for trend			0.9682	0.4252
**Moderate + Strenuous**				
Low	590	360	1.00 (Reference)	1.00 (Reference)
Intermediate	591	339	0.95 (0.82–1.11)	0.97 (0.83–1.12)
High	587	344	0.93 (0.80–1.08)	0.97 (0.83–1.13)
P for trend			0.3187	0.6882
**Recent physical activity**				
**Moderate**				
Low	630	362	1.00 (Reference)	1.00 (Reference)
Intermediate	688	402	1.04 (0.9–1.2)	1.06 (0.92–1.23)
High	450	279	1.08 (0.91–1.27)	1.06 (0.9–1.26)
P for trend			0.3896	0.4535
**Strenuous**				
Low	896	539	1.00 (Reference)	1.00 (Reference)
Intermediate	322	189	1.17 (0.99–1.39)	1.11 (0.94–1.32)
High	550	315	0.93 (0.8–1.08)	0.98 (0.84–1.13)
P for trend			0.4861	0.93
**Moderate + Strenuous**				
Low	598	350	1.00 (Reference)	1.00 (Reference)
Intermediate	589	337	0.96 (0.83–1.12)	1.04 (0.89–1.21)
High	581	356	1.03 (0.88–1.19)	1.06 (0.92–1.24)
P for trend			0.7471	0.4191

a Model 1: adjusted for age at lung cancer diagnosis and the other respective PA variables

b Model 2: adjusted for factors in Model 1 and menopause status with hormonal therapy use, smoking total pack-years by smoking status, and alcohol consumption

**Table 3:** Multivariable hazard ratios (HR) and 95% confidence intervals (CI) for the association between physical activity and mortality among 1,768 women diagnosedthe California Teachers Study stratified by smoking status.

	Never Smokers			Ever Smokers			Former Smokers		
	N	Model 1^a^	Model 2^b^	N	Model 1^a^	Model 2^b^	N	Model 1^a^	Model 2^b^
	Deaths/Cases	HR (95% CI)	HR (95% CI)	Deaths/Cases	HR (95% CI)	HR (95% CI)	Deaths/Cases	HR (95% CI)	HR (95% CI)
**Lifetime PA**									
**Moderate**									
Low	91/188	1.00 (Reference)	1.00 (Reference)	279/423	1.00 (Reference)	1.00 (Reference)	175/282	1.00 (Reference)	1.00 (Reference
Intermediate	85/163	1.16 (0.86–1.57)	1.15 (0.85–1.57)	246/408	0.87 (0.72–1.03)	0.88 (0.74–1.05)	162/272	0.86 (0.69–1.08)	0.84 (0.67–1.05)
High	87/165	1.11 (0.80–1.54)	1.12 (0.80–1.55)	255/421	0.76 (0.62–0.92)	0.77 (0.64–0.94)	146/268	0.64 (0.50–0.82)	0.63 (0.49–0.82)
*P for trend*		0.4805	0.482		0.0058	0.0105		0.0005	0.0004
**Strenuous**									
Low	99/179	1.00 (Reference)	1.00 (Reference)	254/411	1.00 (Reference)	1.00 (Reference)	155/269	1.00 (Reference)	1.00 (Reference
Intermediate	81/171	0.73 (0.54–0.98)	0.69 (0.51–0.94)	271/418	1.18 (0.99–1.41)	1.24 (1.04–1.48)	173/286	1.16 (0.93–1.46)	1.24 (0.99–1.56)
High	83/166	0.71 (0.51–0.99)	0.69 (0.49–0.97)	264/423	1.17 (0.96–1.42)	1.22 (1.00–1.49)	155/267	1.15 (0.90–1.48)	1.18 (0.91–1.53)
*P for trend*		0.0327	0.024		0.0898	0.0312		0.2434	0.1668
**Moderate + Strenuous**									
Low	97/186	1.00 (Reference)	1.00 (Reference)	263/404	1.00 (Reference)	1.00 (Reference)	164/267	1.00 (Reference)	1.00 (Reference
Intermediate	78/162	0.89 (0.66–1.20)	0.80 (0.58–1.09)	261/429	0.98 (0.82–1.16)	1.01 (0.85–1.20)	170/290	1.00 (0.80–1.24)	1.02 (0.82–1.27)
High	88/168	0.89 (0.66–1.19)	0.87 (0.64–1.18)	256/419	0.94 (0.79–1.12)	0.98 (0.83–1.17)	149/265	0.83 (0.67–1.04)	0.83 (0.66–1.05)
*P for trend*		0.4231	0.3792		0.4897	0.8489		0.1139	0.1217
**Recent PA**									
**Moderate**									
Low	81/179	1.00 (Reference)	1.00 (Reference)	281/451	1.00 (Reference)	1.00 (Reference)	152/266	1.00 (Reference)	1.00 (Reference
Intermediate	116/221	1.27 (0.94–1.70)	1.16 (0.86–1.57)	286/467	0.97 (0.82–1.15)	0.97 (0.82–1.15)	190/321	1.00 (0.80–1.25)	0.94 (0.75–1.17)
High	66/116	1.43 (1.02–2.02)	1.28 (0.90–1.81)	213/334	0.93 (0.76–1.12)	0.94 (0.78–1.14)	141/235	0.89 (0.69–1.14)	0.85 (0.66–1.09)
*P for trend*		0.0338	0.1564		0.4355	0.5457		0.3568	0.1865
**Strenuous**									
Low	127/253	1.00 (Reference)	1.00 (Reference)	412/643	1.00 (Reference)	1.00 (Reference)	250/416	1.00 (Reference)	1.00 (Reference
Intermediate	46/90	1.48 (1.04–2.11)	1.62 (1.12–2.34)	143/232	1.03 (0.85–1.25)	1.05 (0.87–1.28)	80/136	1.05 (0.81–1.35)	1.04 (0.81–1.34)
High	90/173	1.03 (0.78–1.38)	1.08 (0.81–1.45)	225/377	0.93 (0.78–1.10)	0.94 (0.79–1.12)	153/270	0.99 (0.79–1.22)	0.97 (0.78–1.21)
*P for trend*		0.7206	0.54		0.4619	0.5988		0.9365	0.862
**Moderate + Strenuous**									
Low	80/166	1.00 (Reference)	1.00 (Reference)	270/432	1.00 (Reference)	1.00 (Reference)	140/246	1.00 (Reference)	1.00 (Reference
Intermediate	90/190	0.91 (0.67–1.23)	0.87 (0.64–1.18)	247/399	1.06 (0.89–1.26)	1.06 (0.88–1.26)	161/270	1.15 (0.92–1.45)	1.11 (0.88–1.41)
High	93/160	1.31 (0.97–1.78)	1.21 (0.89–1.65)	263/421	0.94 (0.79–1.11)	0.95 (0.80–1.13)	182/306	1.04 (0.83–1.30)	0.98 (0.78–1.22)
*P for trend*		0.0862	0.2175		0.4535	0.5381		0.8168	0.7714

a Model 1: adjusted for age at lung cancer diagnosis and the other respective PA variables

b Model 2: adjusted for factors in Model 1 and BMI, education level, and alcohol consumption

## Data Availability

The California Teachers Study data and resources are made available in accordance with the National Institute of Health’s Policy for Data Management and Sharing and the NIH Genomic Data Sharing Policy. The CTS Data Environment, which includes all CTS data, software, and documentation, is open and free of charge to anyone who agrees to and signs the CTS Confidentiality Pledge. Individuals interested in accessing CTS data can sign up for the CTS Researcher Platform and submit a project for feasibility review here: https://calteachersstudy.my.site.com/researchers/s/ The dataset generated and analyzed for the current study are not publicly available as they are housed within the CTS Data Environment but can be made available by the corresponding author on reasonable request.
